# Impacts of Binary Oxide Nanoparticles on the Soybean Plant and Its Rhizosphere, Associated Phytohormones, and Enzymes

**DOI:** 10.3390/molecules28031326

**Published:** 2023-01-30

**Authors:** Titilope Tinu Ajiboye, Timothy Oladiran Ajiboye, Olubukola Oluranti Babalola

**Affiliations:** 1 Food Security and Safety Niche Area, Faculty of Natural and Agricultural Sciences, North-West University, Private Bag X2046, Mmabatho 2735, South Africa; 2 Chemistry Department, Nelson Mandela University, University Way, Summerstrand, Gqeberha 6019, South Africa

**Keywords:** oxide nanoparticles, soybean, microbial community, bioactive compounds, rhizosphere, phytohormones, enzymes

## Abstract

The utilization of binary oxide nanoparticles is geometrically increasing due to their numerous applications. Their intentional or accidental release after usage has led to their omnipresence in the environment. The usage of sludge or fertilizer containing binary oxide nanoparticles is likely to increase the chance of the plants being exposed to these binary oxide nanoparticles. The aim of the present review is to assess the detailed positive and negative impacts of these oxide nanoparticles on the soybean plants and its rhizosphere. In this study, methods of synthesizing binary oxide nanoparticles, as well as the merits and demerits of these methods, are discussed. Furthermore, various methods of characterizing the binary oxide nanoparticles in the tissues of soybean are highlighted. These characterization techniques help to track the nanoparticles inside the soybean plant. In addition, the assessment of rhizosphere microbial communities of soybean that have been exposed to these binary oxide nanoparticles is discussed. The impacts of binary oxide nanoparticles on the leaf, stem, root, seeds, and rhizosphere of soybean plant are comprehensively discussed. The impacts of binary oxides on the bioactive compounds such as phytohormones are also highlighted. Overall, it was observed that the impacts of the oxide nanoparticles on the soybean, rhizosphere, and bioactive compounds were dose-dependent. Lastly, the way forward on research involving the interactions of binary oxide nanoparticles and soybean plants is suggested.

## 1. Introduction

Soybean (*Glycine max* L.) remains one of the most highly cultivated legumes worldwide [1]. In a year, more than 200 million metric tons of soybean are produced, as revealed by the statistic of Food and Agricultural Organization (FAO) in the year 2009 [2]. In 2019, the FAO classified soybean as the fifth largest crop worldwide in terms of agricultural productivity [3]. Soybean is a precursor for biodiesel and biomaterials. Investigations have revealed that it is a good metal accumulator [4], and these numerous uses of soybean have led to it being in high demand. To sustain the high yield of soybean, serious attention has been paid to the availability of various micronutrients in the soil using nanofertilizers. Nanofertilizers (especially those containing oxide nanoparticles) have a high surface area-to-volume ratio and can easily be absorbed by plants [5,6]. These nanoparticles are common in the environment due to their vast applications. They are used as antimicrobial agents, as well as in drug delivery systems, food preservatives, sport equipment, water purifiers, car tire reinforcements, bone reconstruction, and other applications [7,8]. The nanoparticles find their way into the irrigation water and soil, where they interact with the plants.

Exposure of soybean to these nanoparticles can lead to biotransformation, bioaccumulation, translocation, and uptake of the nanoparticles [9]. These processes can have beneficial or detrimental effects on the anatomy and physiology of the soybean plant. For instance, nanoparticles can have negative effects on the growth and germination of plants, and they have the ability to induce phytotoxicity, but they are important in enhancing the performance of crop and seed germination [10]. The understanding of the interaction of the nanoparticles with the plants is, therefore, necessary in understanding the effects of these nanoparticles on plants. Among the nanoparticles that have been largely studied are the binary oxide nanoparticles.

The binary oxide nanoparticles are particularly popular due to their application as photocatalysts and adsorbents in remediating the environment from soil and water pollutants [11,12,13]. Furthermore, they are used as agents for inactivating bacteria in the presence of light [14,15]. Examples of these oxide nanoparticles are iron oxide, titanium oxide, cerium oxide, zinc oxide, silicon oxide, copper oxide, chromium oxide, silver oxide, and aluminum oxide. Investigations have revealed that these oxide nanoparticles bioaccumulate in the soybean plant, and they also translocate into its seeds and leaves [16,17]. Their impacts on the root cannot be overlooked because they have the ability to impair nitrogen fixation, resulting in detrimental effects on the fertility of the soil [18]. The root of plants is the link between the plants and the microbes in the soil. Additionally, it is responsible for transporting water and nutrients to the other parts of the plant. It also secretes exudates such as phenolic compounds, sugars, organic acids, and amino acids [19]. The extent, types, and quantity of these exudates released by the roots are influenced by external factors [20]. One of the factors is represented by the constituents of the rhizosphere of the plants, and binary oxide nanoparticles are often found in the rhizosphere of plants. As far as we know, the impacts of these oxide nanoparticles on the soybean and its environment have not been reviewed. As a result, the aim of the present review was to assess the detailed positive and negative impacts of these oxide nanoparticles on the soybean plant and its rhizosphere, enzymes, and phytohormones. The various characterization tools needed to properly understand the interactions between soybean and these oxide nanoparticles are comprehensively discussed.

## 2. Methods of Synthesizing Binary Oxide Nanoparticles

**Figure 1:**
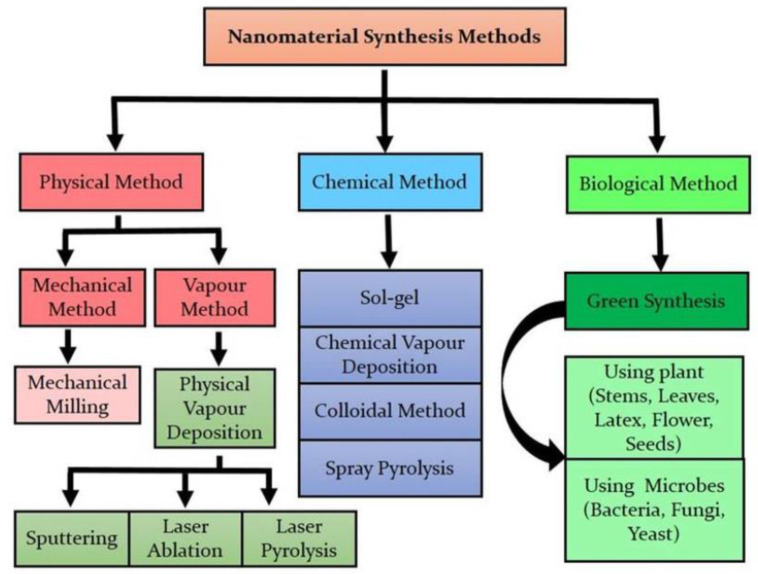
Methods of synthesizing nanomaterials. Reprinted with permission from [29]. Springer Nature (2020).

There are several methods that have been adopted in synthesizing oxide nanoparticles. Each of these methods has its merits and demerits, as summarized in Table 1. In addition to the methods listed in the Table 1, other reported methods are template-assisted precipitation/co-precipitation, flow-injection, electrochemical, oxidation, electron beam lithography/photolithography, and green synthesis [21,22,23,24,25] (Figure 1). Green synthesis is majorly adopted because it is an environmentally friendly, reliable, and sustainable route of synthesis [26]. It can be scaled up to produce nanoparticles on a commercial scale [27]. The byproducts of green synthesis routes are not toxic unlike the byproducts of other conventional methods. Green synthesis requires the use of fungi, bacteria, algae, or plant-derived extracts [26,28].

A particular binary oxide nanoparticle can be prepared using several methods. For example, iron oxides nanoparticles are prepared using physical methods (deposition of gas phase and electron beam lithography), chemical methods (sol–gel, electrochemical, hydrothermal, oxidation, and flow injection), and biological methods (microbial incubation) [30]. Moreover, cerium oxide nanoparticles are prepared using the sol–gel method, hydrothermal and sonochemical methods, spray-drying, template-assisted precipitation, and co-precipitation [31].

**Table 1:** Advantages and disadvantages of methods for synthesizing binary oxide nanoparticles.

Synthetic Method	Brief Description	Advantages	Disadvantages	Ref.
Sol–gel	Preparation via transformation of liquid precursors to sol and finally to a gel structure	Control of morphology is possible by changing the precursors	A toxic organic solvent may be required; processing is associated with contraction	[21,32]
Hydrothermal	Reaction of solid material with aqueous solution at high temperature and pressure	The reaction is usually carried out in a closed system which minimizes pollution; easy to control the nucleation; low temperature required in a suitable solvent; it saves energy	Longer reaction time than techniques such as vapor deposition technique	[24,33]
Sonochemical	The production of nanoparticles using ultrasound under high intensity of sound, high pressure, and high temperature	Possibility of initiating reaction without external agents	Lack of ultrasonic reactors that can produce in commercial quantities	[23,34]
Spray-drying	It involves atomization by using hot drying gas to give dry powder of nanoparticles	Reproducible, fast, and cheap	Reduced yield due to the sticking of the products to the walls of the drying chamber	[35]
Solvothermal	Precursors are stoichiometrically mixed with organic solvent at an elevated temperature to generate nanoparticles	Materials produced have high degree of crystallization	Long time reaction; contamination which requires several washing steps	[24,36,37]
Deposition of gas phase/vapor	Conversion of vapor phase to condensed phase to produce nanoparticles	Thin films of nanoparticles are formed easily	It has high cost and gives low yield	[21,25]
Mechanical/ball milling	Employing impacts from mechanical energy to generate inorganic materials	Cheap, easy to optimize, and gives pure product	Contamination is possible; it requires a long time; high energy is required	[28]
Microwave	Utilizing microwave irradiation to raise the temperature of reactants in solution leading to the formation of nanoparticles	Easy to reproduce; short reaction time needed; high yield is obtained	High synthetic cost; commercialization is tedious	[24]
Laser/spray pyrolysis	Laser beam is used to heat up or decompose the precursor leading to the formation of nanoparticles	Relatively cheap; morphological modulation is possible	The reactors needed for pyrolysis are expensive	[21]

## 3. The Methods of Characterizing Binary Oxide Nanoparticles Inside Soybean Plant

**Figure 2:**
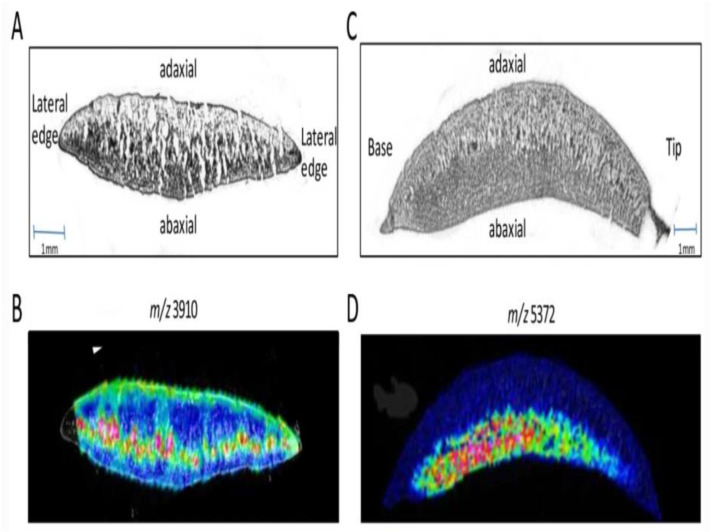
MALDI imaging in soybean cotyledons. (**A**) A cross-section of the cotyledon, exposing the center midway between the tip and the base. (**B**) MALDI-MSI cross-section showing the peak intensity and localization for m/z 3918. (**C**) Cross-section of the cotyledon cut axially from tip to base. (**D**) MALDI-MSI cross-section showing the peak intensity and localization for m/z 5372. Reproduced from [39]; Springer Nature(2011); open access under Creative Commons Agreement.

Different characterization techniques have been used to test the viability of different parts of soybean plants. One of the techniques used for characterizing the seed of soybean plant is X-ray. This technique can detect the mechanical and stink bug damage in soybean plant [38]. It is possible to know if healthy or unhealthy seedlings of soybean will be produced through this technique. Another technique that can give the distribution of biomolecules in soybean is time-of-flight mass spectrometry imaging coupled with matrix-assisted laser desorption ionization (MALDI-MSI). To use this technique, a particular area is spotted on the tissue of soybean, and alignment software is used to compare its protein profile [39]. Examples of images obtained via this technique are shown in Figure 2. Just like there are analytical tools for characterizing various parts of soybean plants, the nanoparticles are also characterized by various analytical tools. Each of these techniques has unique applications and principles of operation. Some of these techniques, as well as their applications and principles of operation, are summarized in Table 2.

**Figure 3:**
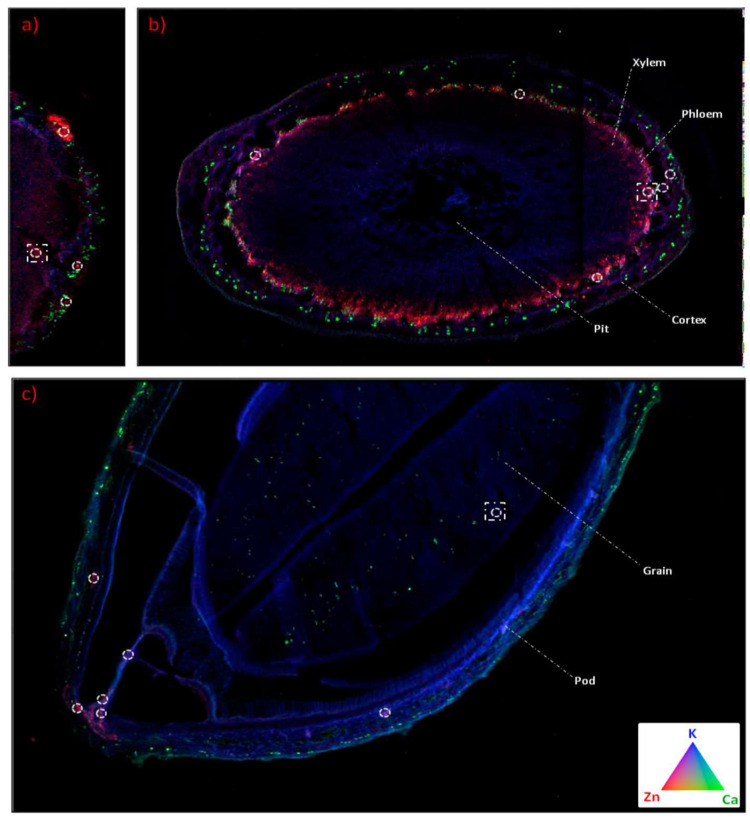
Tricolor μ-XRF maps (blue = K, green = Ca, and red = Zn): (**a**) nodule, (**b**) stem, and (**c**) pod maps. White circles mark the areas with high intensity of Zn, according to μ-XANES. Reproduced with permission from [40]; copyright (2013) America Chemical Society.

The transportation of nanoparticles inside the soybean plants is also possible using some of the analytical techniques. One of the techniques used is synchrotron micro-X-ray fluorescence (XRF). Hernandez-Viezcas et al. used μ-XRF and μ-XANES to track both CeO_2_ and ZnO in soybean plants. Through these techniques, it was possible to determine if there are residual nanoparticles in any part of the soybean plants, and to evaluate the interaction of soybean plants with the oxide nanoparticles [40]. Examples of images obtained in tracking ZnO and other nutrients (calcium and potassium) through these techniques are shown in Figure 3. Inductively coupled plasma atomic emission spectrometry (ICP-OES), inductively coupled plasma mass spectrometry (ICP-MS), radiotracer and autoradiograph, μ-XAS, are micro-particle-induced X-ray emission are specific examples of these methods for tracking nanoparticles in different plants, as reviewed in [41]. In fact, methods that can be used to detect the presence of microbes in plants are impacted by nanoparticles. One such method is matrix-assisted laser desorption/ionization time-of-flight mass spectrometry (MALDI-TOF MS) [42].

**Table 2:** Methods of characterizing binary oxide nanoparticles.

Characterization Tools	Application	Principle of Operation	Ref.
X-ray diffraction (XRD)	To determine the dimensions of the lattice, particle size and crystallinity; it is also used for crystal characterization	The interaction of a light having a single wavelength with the oxide nanoparticles.	[43,44]
UV/visible absorption spectroscopy	To determine the stability of the oxide nanoparticles and for identification purposes	The plot of coefficient of extinction against wavelength obtained when light of known intensity passes through the sample to the detector	[45]
Fourier-transform infrared spectroscopy (FTIR)	To determine the functional group of molecules attached to the oxide nanoparticles	Interaction of infrared radiation causing vibration and interaction of molecules	[46]
Dynamic light scattering (DLS)	To measure the distribution of the particle size in colloid or suspension	Detection of the scattered light at a known angle after the sample is focused with a laser beam	[47]
Scanning electron microscopy (SEM)	To determine the surface images of oxide nanoparticles	Interaction of the electrons in the sample with the beam of electron from the machine to generate captured signals	[48,49]
Transmission electron microscopy (TEM)	To determine the morphology, size, and internal morphology of oxide nanoparticles	The beams of electrons pass through the oxide nanoparticles; the beam is scattered, while the lens captures the scattered electrons to form an image	[50]
Energy-dispersive X-ray analysis (EDAX)	To determine the elements that are present in oxide nanoparticles	Electrons are knocked off from the inner shell of electrons when it is bombarded with a beam of electrons, leading to the generation of a positively charged hole which takes up another electron from the valence shell due to electrostatic forces of attraction	[48,49]
X-ray phosphorescence (XPS)	To determine the purity of oxide nanoparticles	Bombardment of nanoparticles with high energy radiations to give a characteristic fluorescent emission.	[51]
Atomic force microscopy (AFM)	To determine the volume distribution, surface area, roughness, morphology, and size of oxide nanoparticles	A micro-cantilever is used with the side having weaker force contacting the sample; the fluctuation of the probe is measured	[52,53,54]
Thermal gravimetric analysis (TGA)	To determine the stability of oxide nanoparticles under heat	The change in weight is plotted as a function of temperature	[55]
Dynamic light scattering (DLS)	To determine the state of aggregation of oxide nanoparticles	It operates on the basis of “Brownian motion”	[56]

## 4. Effects of Binary Oxide Nanoparticles on Soybean Plant

Nanoparticles have been found to have significant effects on the germination rate of plants. The nanoparticle is small, and it assists the penetration of nutrients and water into the seed of soybean without altering its internal structure [57]. Every part of the soybean plant is affected by the presence of oxide nanoparticles. In fact, the rhizosphere is not exempt. Nanoparticles can influence the growth and germination of soybean plants. They can prevent the plant from going moldy and cause an increase in water utilization and the absorption properties of plants [58]. This section explains the impact of oxide nanoparticles on various parts of the soybean and its rhizosphere, enzymes, and hormones.

### 4.1. Effects of Binary Oxide Nanoparticles on the Rhizosphere Microbial Community of Soybean Plant

The region surrounding the roots of plants, usually 2–80 mm from the root depending on the species of plant, is called the rhizosphere, which is rich in enzymes, microbes, and nutrients [59,60]. The microorganisms found in the rhizosphere of soybean form part of the ecological feeding system in the rhizosphere by using the root-exuding chemicals which help to regulate the activities that go on in the soybean root’s attached soil. The final microbial assemblage and composition in the rhizosphere are a result of the rich species of microbes found in soybean’s bulk soil [61]. Researchers have reported two theories (neutral theory and niche theory) to explain the reason for microbiome assemblage in the rhizosphere [62]. The neutral theory is a sampling, dispersal-assembled, and stochastic theory based on the interaction and origin of a biological population. It assumes that the interactions are operated on individual levels [63]. The niche theory, on the other hand, is based on the spatial homogeneity of the local environment [64].

The microbes that are present in the rhizosphere are known as rhizosphere microbial communities. Degradation of soil organic carbon, cycling of nitrogen, and mineralization of nutrients are all biogeometrical functions of the microbial communities of the soil [65]. Hence, the availability of organic carbon and nutrient depends on the state of the microbial communities. The assessment of microbial communities of useful plants such as soybean is necessary [66]. It has been reported that the toxicity of oxide nanoparticles on the microbial communities is a function of the specific microbes that are present in the rhizosphere [67] and the type of soil under investigation [66]. Some oxide nanoparticles show no effect on the rhizosphere bacterial communities in an uncultivated soil. However, the bacterial communities of soybean-cultivated soil are altered.

Ge et al. [68] observed that application of 0.5 g/kg of zinc oxide nanoparticles increased the Azotobacter, Clostridium, Rhodospirillaceae, and Ensifer in the soybean cultivated soil, while the population of Sphingomonas and Rhizobium significantly decreased. This same effect was observed with cerium oxide nanoparticles, but a lower quantity of cerium oxide was used (0.1 g/kg). The effect of CeO_2_ was particularly observed in the soil cultivated with soybean unlike the unplanted soil, where bacterial communities were not affected by CeO_2_ nanoparticles. A large quantity of ZnO was required because soybean root takes up zinc ions, thereby reducing its availability and effects on soil microbial communities [68]. The reduced availability of ZnO can also be as a result of the carbon that is released by root exudates, which causes immobilization of these oxide nanoparticles. The impact of oxide nanoparticles on the growth of A. vinelandii has also been investigated. It was observed that there was no effect on the growth of A. vinelandii when TiO_2_ was used for the investigations. However, ≤10 mg/L tungsten oxide displayed a significant toxic effect on the microbe. The toxic effect of tungsten oxide was linked to catechol–metallophore interference [69].

TiO_2_ has impacts on the rhizospheric microbial community, whether it is doped or undoped. For instance, even at low concentrations of nitrogen doped TiO_2_ and undoped TiO_2_ nanoparticles, the communities of mycorrhizal fungi and other prominent functional groups in the rhizosphere of soybean are affected. These alterations in the microbial communities may influence the growth of soybean plant, especially in the absence or insufficiency of nutrients and moisture [67]. Since arbuscular mycorrhizal fungi have the ability to form a symbiotic relationship with the root of plants, the ability to acquire nutrients in the root of Trifolium repens and other arbuscular mycorrhizal fungi may be affected, and the content of the glomalin may be significantly reduced [70]. SiO_2_ and TiO_2_ increased the activity of reductase in soybean, and they also activated the antioxidant defense system [58,71].

### 4.2. Effects of Binary Oxide Nanoparticles on the Leaf of Soybean Plant

Inorganic iron has low solubility under a near neutral pH, which causes a reduction in the concentration of iron in the soil solution. Deficiency of iron in plants leads to a reduction in chlorophyll biosynthesis and iron deficiency chlorosis [1,2]. This in turn slows the growth rate of soybean plant and yellowing of leaves. Since iron plays an important role in photosynthesis, there is a need to introduce an alternative iron source for better leaf (and chlorophyll) development in soybean. Iron oxide nanoparticles are among the sources of iron used for this purpose. For instance, the introduction of iron oxide into soybean plant under hydroponic conditions led to enhanced chlorophyll levels without any noted detrimental effects on the plants [72]. Even when iron oxide nanoparticles were coated with fulvic acid, there was still an enhancement of chlorophyll content of soybean plants [73].

The introduction of TiO_2_ nanoparticles to cultivated soybean in a cadmium stress environment led to a decrease in the chlorophyll content of the plant. The reduction in chlorophyll content was as a result of disturbance of the enzyme in charge of chlorophyll synthesis [74]. In fact, there was also a reduction in leaf carbon content when TiO_2_ was introduced to soybean, and the reduction was significantly greater than when Fe_3_O_4_ nanoparticles were used instead of TiO_2_ [75]. Another oxide nanoparticle that has been used to investigate the effect of oxide nanoparticles on chlorophyll is Cr_2_O_3_. There was damage to the chloroplast thylakoid structure and ultrastructure, leading to the alteration of photosynthetic system. Inhibition of the activities of electron acceptor NADP+ was also observed [76].

Stowers et al. examined the effect of exposure of soybean to cerium oxide nanoparticles. These researchers discovered that the rate of photosynthesis of soybean seedlings planted in sterilized soil was 122% better than that of those planted in unsterilized soil, when 100 mg/kg of this nanoparticle was used. On the contrary, the rate of photosynthesis dropped in sterilized soil by 67.2% when the quantity of cerium oxide was increased from 100 mg/kg to 500 mg/kg [59], which showed that the photosynthetic rate of soybean varies with the amount of cerium oxide nanoparticles present in soybean. The ratio of chlorophyll a to chlorophyll b is also influenced by the presence of cerium oxide nanoparticles. The ratio of chlorophyll a to chlorophyll b in an unsterilized soil loaded with 500 mg/kg cerium oxide nanoparticles increased from 1.94 to 3.05 in an unsterilized soil. On the contrary, the ratio remained constant at 2.55 when the soil was sterilized [59]. The development of the trifoliate leaves of soybean is also affected by the presence of oxide nanoparticles. For instance, after 14 days of cultivation of soybean in the presence of 500 mg/kg zinc oxide nanoparticles, there was inhibition in the formation of first trifoliate compared with the control. However, there were minimal effects on the leaves when 50 mg/kg of the zinc oxide nanoparticles were introduced into the system [9].

### 4.3. The Effects of Binary Oxide Nanoparticles on the Stem of Soybean Plant

Spots, etiolation, and early wilting were observed on the stem of soybean exposed to 500 mg/kg of zinc oxide nanoparticles. However, there was no significant difference with the control when 50 mg/kg of the nanoparticle was used [9]. This observation showed that the amount of zinc oxide nanoparticle determines its effect on the stem of soybean plant. A similar effect was observed when zinc oxide nanoparticles were introduced into the soil during the cultivation of another leguminous plant (Mungbean seed) [77].

**Figure 4:**
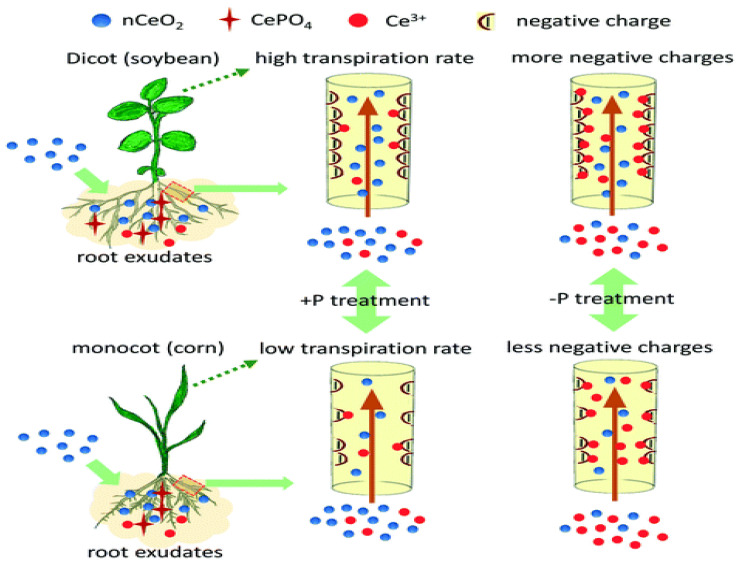
Comparative studies of translocation of cerium oxide nanoparticles and cerium ions on corn (monocot) and soybean (dicot). Reproduced from [81]; Royal Society of Chemistry (open access under Creative Commons Agreement).

The low solubility coupled with the high stability of cerium oxide in the environment, contributes to its availability in the plants [78]. Cerium oxide nanoparticles caused a reduction in the stem size of soybean plant [79]. This was due to the pronounced interaction between the cerium oxide and soybean plant, as shown by the total cerium concentration in the soybean plant [80]. The type of soil used and the concentration of cerium oxide influence the amount of cerium that accumulates in the tissue of soybean plant [59]. Soybean translocates and transforms cerium more than corn under the same conditions. Its ability to translocate and transform cerium is linked to the chemistry of the surrounding solution. Additionally, the composition of roots exudates and xylems also determines the extent of cerium oxide transformation in soybean plants [81]. The rate of transpiration of cerium oxide nanoparticles by soybean is also influenced by the presence of phosphorus, the structure of the xylem, and the zeta potential, as shown in Figure 4.

Aluminum oxide nanoparticles also have noticeable impacts on the stems of soybean. The presence of aluminum oxide nanoparticles did not increase the length of the stems; however, it was reported to increase the activities of peroxidase around the cell wall while reducing the activities of the enzymes that break down ammonia and phenylalanine in the stem of soybean. Unlike in the root of soybean, the presence of aluminum oxide nanoparticles increased the content of parahydroxyphenyl in the stem. Phenolic compounds also increased in the stem of soybean as a result of aluminum oxide nanoparticles. On the contrary, the amount of paracoumaric acid in the stems of soybean reduced due to the presence of aluminum oxide [82].

**Figure 5:**
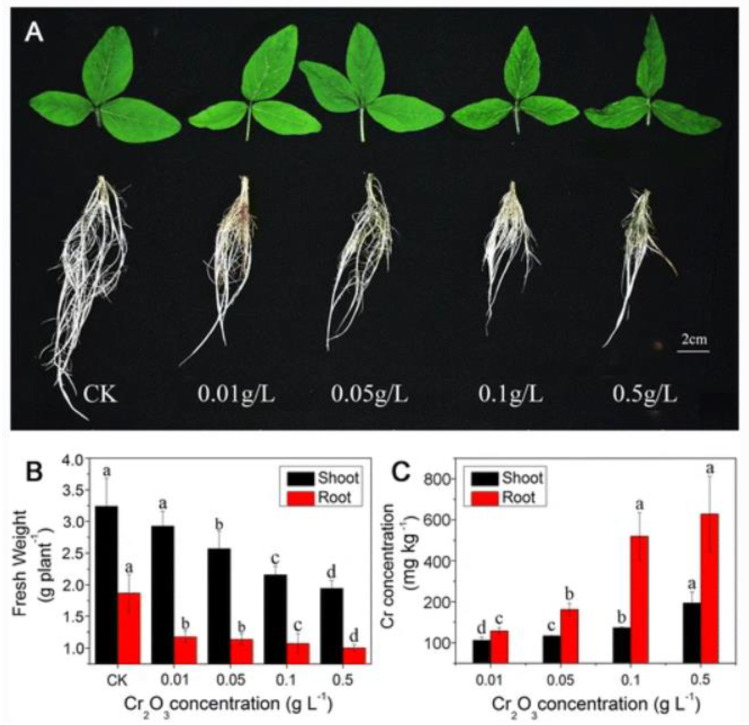
Effects of chromium nanoparticles treatment on (**A**) the growth of shoots and roots of soybean (**B**) the biomass of soybean after 14 days of the nanoparticles treatment (**C**) the residual contents of chromium in the roots and shoots after 14 days of nanoparticle treatment. Bars with different letters are significantly different at *p* < 0.05. Reprinted with permission from [76]; copyright (2013), Springer Nature.

There was a 140% increase in the shoot biomass compared to control with the introduction of 60 ppm of CuO nanoparticles [83]. However, when the concentration of CuO nanoparticles was increased to 2000 ppm, there was a 100% decrease in the shoot biomass, revealing that the optimum concentration beneficial to soybean is 60 ppm, whereas a high concentration of this same nanoparticle has detrimental effects on soybean plants. On the contrary, there was an observable decrease in the weight of the shoot when Cr_2_O_3_ nanoparticles were used in another investigation. The decrease in biomass observed for the shoot was lower compared with what was observed in the root. In particular, there was a 46.3% biomass reduction in the root, whereas there was a 9.9% biomass reduction in the shoot of the soybean plant [76]. The reduction in biomass varied with the concentration of Cr_2_O_3_ nanoparticles, as shown in Figure 5.

### 4.4. The Effects of Binary Oxide Nanoparticles on the Root of Soybean Plant

**Figure 6:**
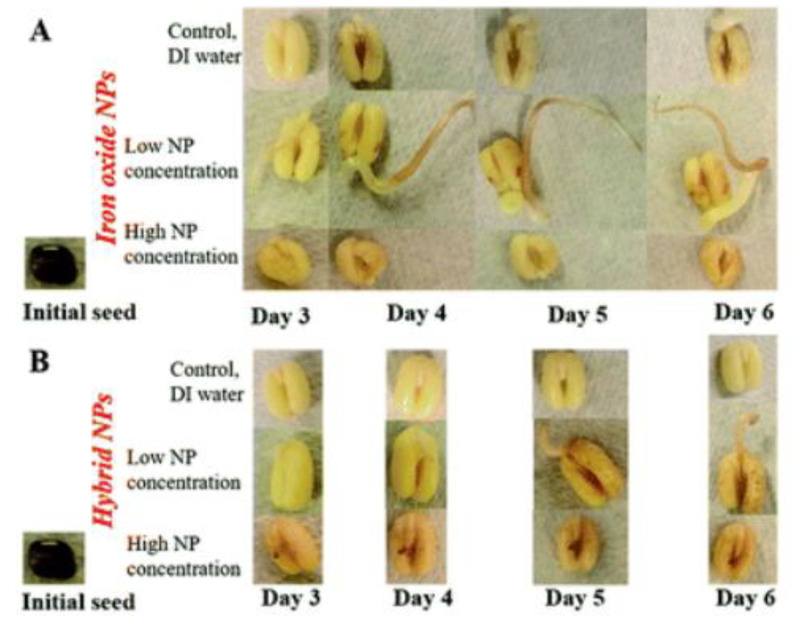
The growth of seedlings planted with nanoparticles at neutral pH: (**A**) legume root grown in the solution of iron oxide nanoparticles; (**B**) legume root planted in platinum-functionalized nanoparticles. High nanoparticle concentration = 27.7 mg/L Fe; low nanoparticle concentration = 5.54 × 10^−3^ mg/L Fe. Reprinted from [85]; Royal Society of Chemistry (Open Access under Creative Common Agreement).

It has been reported that, when nanoparticles are applied to the soil, they are concentrated in the root tips and root hairs of plants. If the nanoparticle is iron oxide, the ratio of divalent iron to the total iron is boosted with a high biomineralization value [84]. Iron oxide nanoparticles have shown a significant impact in the root development of legumes. This was particularly obvious in the formation of root nodules [73]. The chemical composition, morphology, and concentration of iron oxide nanoparticles played an important role in the growth of the roots of leguminous embryos. A low concentration of iron oxide nanoparticles (∼5.54 × 10^−3^ mg/L Fe) was better than a high concentration in ensuring better seedling growth. Metallic-doped iron oxide nanoparticles (hybrid NPs) performed worse than undoped iron oxide nanoparticles [85], as illustrated in Figure 6.

The presence of zinc oxide nanoparticles in the soil during cultivation has an inhibitory effect on the root anatomy of the soybean. Treatment of the soil used for planting soybean with 500 mg/kg of zinc oxide nanoparticles led to an 88% reduction in the surface area of the root and an 87% reduction in the volume of root compared to the control, which was planted without zinc oxide nanoparticles. Furthermore, the treated soybean had fewer root hairs compared to the control [9]. On the contrary, presoaking of soybean seeds with zinc oxide nanoparticles under saline soil conditions led to stimulation of the growth parameters of soybean. There was also a significant increase in the level of MDA and proline when 50 mg/L of the zinc oxide was used compared with the control. The improvement in the growth of soybean under this condition was attributed to the presence of zinc oxide nanoparticles because there was a 65% reduction in the germination rate when soybean was planted in saline soil without the seed being soaked with zinc oxide nanoparticles [86]. This highlights the fact that zinc oxide nanoparticles can have both a positive and a negative impact on soybean plants depending on the method of application.

There is higher rate of absorbance of cerium oxide nanoparticles in the root of soybean than in its shoot. When concentrated in the root, cerium oxide nanoparticles increase the growth of the root of soybean [79]. The amount of root nodules produced by soybean plant is influenced by the presence of cerium oxide nanoparticles. This impacts the rate at which nitrogen is being taken up by the soybeans [80]. Aluminum oxide nanoparticles induce crack formation close to the apex of the root of soybean, as well as damage the root cap. They interact with some organelles in the cell wall and cytosols of the root. Hence, they are found to concentrate in these parts of soybean roots. When they are in the root, they cause the content of lignin to be boosted in the root. Furthermore, they increase the phenolic, ferulic, and paracoumaric acid contents of soybean roots [82]. Increased lignification of the cells in the root of soybean plant has been reported as a result of its exposure to CuO nanoparticles. The increased lignification slows down the root development of soybean seedlings. Moreover, the generation of peroxidase and hydrogen peroxide is significantly higher in the presence of CuO nanoparticles [3]. Application of 60 ppm of CuO nanoparticles led to an increase in root biomass by 177% compared to the control [83]. Root necrosis and inhibition of elongation of the root of soybean plants as a result of exposure to CuO nanoparticles have also been reported [87].

### 4.5. The Effects of Binary Oxide Nanoparticles on the Seeds of Soybean Plant

The nutrient availability in the pods and seeds of soybean are affected by the presence of oxide nanoparticles. This alteration of nutrients depends on the concentration and the type of oxide nanoparticle impacting the soybean. For example, with a low concentration of cerium oxide nanoparticles, the amount of sodium in the seed pod is reduced, whereas, at a high concentration of cerium oxide, the amount of calcium is reduced, while the amounts of copper and phosphorus are increased. When zinc oxide is introduced, the amounts of copper, manganese, and zinc increase in the seed pods [88]. In another study, treatment with 500 mg/kg of zinc oxide nanoparticles caused reductions in the shoot and root of soybean. Additionally, the seed did not form at all due to the negative impact of zinc oxide nanoparticles on the seed formation of soybean [9]. Conversely, there was 100% seed germination when CuO nanoparticles were introduced into the soybean during cultivation. Normal seed germination may have been a result of the presence of the seed coat, which protected the embryo from the effect of the nanoparticles [87]. In another investigation, the size of the CuO nanoparticles was found to influence the yield of soybean seed obtained [3].

### 4.6. The Effects of Binary Oxide Nanoparticles on Phytohormones and Enzymes of Soybean Plant

The exposure of soybean to nanoparticles could lead to oxidative stress conditions, where reactive oxygen species such as hydroxyl radicals, hydrogen peroxide, singlet oxygen, and superoxide radicals are generated. For oxidative stress not to occur, the level of reactive oxygen species is controlled using antioxidants. Some of these antioxidants are enzymes such as superoxide dismutase [89]. Phytohormones are molecules generated inside the plants to either mediate or regulate the growth and development of plants by altering the process of metabolism. Examples of phytohormones are peptide hormones, jasmonates, cytokinnins, auxins, ethylene, giberrellins, brassinosteroids, and abscisic acid [90]. Some of these phytohormones perform more than one function in plants, and they can be used to assess the toxicity of nanoparticle on soybean plant [90]. Zinc oxide nanoparticles stimulate the formation of metabolites, enzymes, antioxidants, osmolytes, and phytohormones, when used in an appropriate dosage. They can also be used to combat abiotic stress in soybean [91]. Foliar application of CuO nanoparticles has also been used to modulate the status of phytohormones in soybean plants, leading to significant reversal of the damage to the photosynthetic leaves and biomass of soybean plants [92].

Lipid peroxidation is often measured by measuring the accumulation of malondialdehyde in the tissues of plants [93]. Both the size and the dosage of CuO nanoparticles were found to have pronounced effects on the malondialdehyde level, with the highest accumulation recorded when 250 mg/kg of the nanoparticles were used. It was reported that there was maximum hydrogen peroxide production when 500 mg/kg of CuO was applied, in contrast to when a lower dosage of the nanoparticles was used for the similar task. CuO nanoparticles also have an impact on the activities of enzymes such as superoxide dismutase, catalase, and guaiacol peroxidase. The impacts of CuO nanoparticles on these enzymes were found to be dependent on the dosage and particle size of the nanoparticles [3]. Generally, investigations on the bioactive exudates from soybean are not possible without the use of separation techniques such as high-performance liquid chromatography (HPLC), Sephadex chromatography, flash chromatography, column chromatography, and thin-layer chromatography (TLC). Moreover, nonchromatographic techniques can be applied, such as Fourier-transform infrared spectroscopy (FTIR), phytochemical screening assay, and the use of monoclonal antibodies (typically called an immunoassay) [94]. Other rapid techniques for determining the bioactive compounds in soybean plants before and after their exposure to the oxide nanoparticles have been reported. For instance, Chien et al. [95] developed the sequential window acquisition of all theoretical fragment ions (SWATH) technique coupled with mass spectrometry for the determination of various bioactive compounds in soybean plants.

## 5. Metagenomics as a Tool for Identifying Microbiomes

A detailed understanding of microbial structure and functions is necessary because microbes form mutualistic and beneficial relationships with most plants [96]. Metagenomics is a useful tool for investigating the microbial diversities of microbial communities. This tool requires the extraction and cloning of DNA to understand the pool of genome organisms [97]. This technique helps in establishing the taxon, as well as functions of the established taxon, and in identifying the gene and assemblage of the genomes of microorganisms in the environment. Researchers have explained the importance of not using only one sequencing technique to properly study microorganisms that are not abundant in a community containing many microbiomes [98,99]. Krishnamoorthy et al. established the use of metagenomics in showing structural diversity and unestablished functions of microbiomes [100].

Metagenomics deals with the collection of genomes of microbes to determine the diversity and ecology of the microbes in the rhizosphere, as well as the functional potentials of the microbiomes in the environment [101]. This technology has been used to assess different microbial communities in different plants. For instance, Sugiyama et al. [102] investigated the physiological properties of bacterial communities in the bulk and rhizospheric soil and roots of soybean through the use of a metagenomic approach. The technique has also been used to obtain detailed information on specific rhizospheric microbes [103] and to understand the role, number, and composition of these microbes [104,105].

### Metagenomics as a Tool for Investigating the Microbiome of Soybean Rhizosphere

The environment of the rhizosphere is likely to be richer than that of the bulk soil since the bulk soil usually experiences less chronic stress compared to the rhizosphere [101]. To properly describe the nature of the plant–soil interface, the knowledge of the user interactions among the microorganisms present in the rhizosphere is necessary [106]. To understand this beneficial interaction and microbial diversities, molecular techniques such as fluorescent in situ hybridization (FISH) [107], terminal restriction fragment length polymorphism (T-RFLP) [108], restriction fragment length polymorphism (RFLP) [109], denaturing gradient gel electrophoresis (DGGE and TGGE) [110], cloning and sequencing of ribosomal genes, and polymerase chain reaction (PCR) [111] are used. In these techniques, the 16S rRNA gene is used as a phylogenetic marker to analyze microbial diversities because this gene is conserved through numerous years of evolution [112]. Large amounts of nucleotide data are generated from most of these techniques. Therefore, bioinformatics software such as the Phylogenetic Investigation of Communities by Reconstruction of Unobserved States (PICRUSt), PacBio, Computer-Aided Room Analyzer (CARMA), Metagenome Analyzer (MEGAN), and Quantitative Insights into Microbial Ecology (QIIME) **[101]** is used. Platforms such as Metagenomics Rapid Annotation using Subsystem Technology (MG-RAST) [113] and Cloud Virtual Resource (CloVR) [114] are then used for microbial diversity analysis [101].

Slattery et al. [115] successfully used metagenomics to investigate the effect of cerium oxide on the microbiome of soybean. This group discovered that a low concentration of nanoparticles in the soil can modulate the biological system. Furthermore, metagenomics showed that incubation of the nanoparticle in the natural soil can have opposite effects on soybean compared to when cerium oxide nanoparticles are used without incubation. Another discovery, with the metagenomics tool by Slattery et al., is that the aging factor of the cerium oxide nanoparticles influences the soil rhizosphere microbiome community. In addition to these findings, metagenomics, along with other analytical equipment, has been used to understand the effects of root exudates on the microbial community of plants [116]. It has been discovered that the relative abundance of some plant growth-promoting rhizobacteria is enhanced, while cerium oxide nanoparticles in the plant’s rhizosphere lead to a reduction in the microbial diversity of the rhizosphere [116]. All these discoveries could not have been possible without the use of metagenomics tools. Metagenomics has also been a useful tool for understanding the functional and taxonomic levels that exist in the rhizosphere community of soybean. For instance, Mendes et al. [62] observed that rhizosphere microbial communities undergo a niche-based process influenced by environmental factors and the selection power of soybean plants.

## 6. Conclusions and Future Perspectives

The impacts of different oxide nanoparticles on the rhizosphere, roots, stems, seeds and leaves of soybean plants were reviewed. Generally, they display both detrimental and beneficial effects on soybean plants. Most of these oxide nanoparticles commonly showed beneficial effects when used controllably at a low dosage, whereas, at a high dosage, they generated stress or toxicity, leading to the disruption of metabolism on the cellular level. Hence, there is an urgent need to study the optimum dosage of these oxide nanoparticles that would be beneficial for soybean cultivation. Furthermore, the effect of the synthesis method of binary oxide nanoparticles on the physiology and anatomy of the soybean plant should be studied. Looking ahead, the use of oxide nanoparticles to control the viral disease of soybean should be investigated. Some of the viral diseases of soybean plants that can be investigated are alfalfa mosaic virus, peanut stunt virus, peanut mottle virus, soybean dwarf virus, soybean vein necrosis virus, bean pod mottle virus, and soybean mosaic virus. Furthermore, the impact of different heterojunction systems and compositions of oxide nanoparticles on soybean plants has not been studied. Efforts should be geared toward investigating the impacts of ternary oxide nanoparticles and carbon-based materials (such as cyclodextrin, graphene, and graphene oxide) on soybean and other plants. Another question that researchers should try to answer is how different soil types affect the binary oxide nanoparticles in the tissues and rhizosphere of soybean plants. The effects of binary oxide nanoparticles on soybean metabolites such as saponin and isoflavone should be studied with detailed characterization. Lastly, studies on the effects of oxide nanoparticles on other leguminous and non-leguminous crops should be investigated.
